# Synthesis and Pharmacokinetics of Nanosized NH_2_‑UiO‑66 (Zr) Metal-Organic Frameworks

**DOI:** 10.1134/S1607672925601477

**Published:** 2026-01-12

**Authors:** A. B. Mirkasymov, V. I. Rodionov, D. A. Pokhorukov, O. Yu. Griaznova, N. K. Ivshina, I. V. Lunyov, I. V. Zelepukin, S. M. Deyev

**Affiliations:** 1 Shemyakin-Ovchinnikov Institute of Bioorganic Chemistry, Russian Academy of Sciences, 117997 Moscow, Russia; 2 National Research Nuclear University MEPhI (Moscow Engineering Physics Institute), 115409 Moscow, Russia

**Keywords:** metal-organic frameworks, pharmacokinetics, nanoparticles, drug delivery

## Abstract

The application of porous nanomaterials in drug delivery offers a promising strategy to mitigate the adverse side effects of chemotherapy. In this study, we report the synthesis of nanosized NH_2_-UiO-66 (Zr) metal-organic frameworks as carriers of doxorubicin. The nanoparticles exhibited high crystallinity with an average size of 44 nm. Surface functionalization with polyethylene glycol (PEG) markedly enhanced their colloidal stability under physiological conditions. Coated NH_2_-UiO-66 (Zr)@PEG particles demonstrated prolonged circulation in the bloodstream and a significant reduction of nonspecific accumulation in organs with high vascularization. Importantly, these particles retained their capacity for doxorubicin loading, highlighting their potential for drug delivery.

## INTRODUCTION

Doxorubicin demonstrates high efficacy in the treatment of various malignant neoplasms, including breast cancer and lymphoma. However, its clinical application is limited by adverse effects, including cardiotoxicity and hematopoietic suppression [1]. This governs development of novel approaches for targeted drug delivery to tumors. One promising direction is the use of nanoparticles as drug carriers, that have the ability to accumulate in tumors via enhanced permeability and retention effect (EPR effect) [2] and perform controlled drug release in tumour microenvironment.

Metal-organic frameworks (MOFs) are attractive candidates for drug delivery due to their high surface area, regular porosity, and structural variability [3]. For instance, MOFs with UiO-66 (Zr) crystal type have large octahedral (∼11 Å) and tetrahedral pores (∼8 Å), a surface area greater than 1000 m^2^/g, and chemical stability over a wide range of pH, facilitating loading of various drugs [4]. UiO‑66 (Zr) has been considered for the loading and controlled release of doxorubicin due to having a coordination interaction between the Zr (IV) clusters and hydroxyl groups of the drug [5]. High loading has also been demonstrated for cisplatin (12% by particle weight) and 5-fluorouracil (27%) [6, 7].

However, UiO-66 (Zr) MOFs do not have colloidal stability under physiological conditions due to the association of Zr(IV) clusters with phosphate anions [8, 9]. Aggregation of nanoparticles in the bloodstream induces their nonspecific accumulation in the capillaries of the lungs, while increase of the size of nanoparticles promotes recognition by macrophages of the mononuclear phagocyte system [10, 11]. Therefore, to improve the pharmacokinetics of UiO-66 (Zr), it is necessary to develop methods for their colloidal stabilization.

In this study, nanoscale NH_2_-UiO-66 (Zr) MOFs were synthesized, the crystallinity of nanoparticles was confirmed by X-ray diffraction analysis. We modified the surface of the nanoparticles with polyethylene glycol and demonstrate possibility to load them with doxorubicin for potential chemotherapy. The coated particles retained long-term colloidal stability under physiological conditions. In addition, the coating prolonged the circulation of nanoparticles in the bloodstream and influenced their biodistribution in healthy mice, with a significant reduction in nonspecific uptake in the lungs and spleen. This study is an important for developing drug delivery systems based on NH_2_-UiO-66 (Zr) metal-organic frameworks.

## MATERIALS AND METHODS

NH_2_-UiO-66 (Zr) nanoparticles were synthesized by a hydrothermal method with heating by microwave irradiation [12]. For this, 0.5 mmol of ZrCl_4_ was mixed with 0.5 mmol of 2-aminoterephthalic acid in 4 mL of dimethylformamide. Then, 0.84 mL of glacial acetic acid and 54 μL of distilled water were added to the mixture. The reaction mixture was heated at 110°C for 15 min in a Monowave 200 reactor (Anton Paar, Austria). The crystalline particles were separated from unreacted compounds by successive centrifugation in dimethylformamide, ethanol, and water (12 000 g, 10 min). The nanoparticles were coated with silane-polyethylene glycol (5 kDa) using the Stöber method [8]. For this, 1 mg of particles was mixed with the polymer at a concentration of 10 g/L in water and heated at 70°C for 30 min. To remove unbound polymer, the particles were washed in water by centrifugation (16 000 g, 7 min).

The crystalline structure was examined by X-ray diffraction (XRD). The powder diffraction pattern of the nanoparticles was recorded in the 2θ range of 5–50° using a Malvern PANalytical X’pert Pro MPD diffractometer with a CuKα radiation source (λ = 1.540 Å) at an operating voltage of 45 kV and a current of 40 mA. Electron micrographs of the nanoparticles were obtained by scanning electron microscopy (SEM) using a MAIA3 microscope (Tescan, Czech Republic) at an accelerating voltage of 20 kV. Particle size distribution was analyzed with ImageJ 1.8.0 software by measuring at least 500 particles. Hydrodynamic size and ζ‑potentials were measured using a Malvern Zetasizer Nano ZS device (Malvern Instruments, UK). Experiments were performed in distilled water or phosphate-buffered saline (PBS) for hydrodynamic size analysis, and in 10 mM NaCl for ζ‑potential analysis.

For doxorubicin loading into NH_2_-UiO-66 (Zr)@PEG nanoparticles, 5 μg of particles were mixed with 5 μg of doxorubicin in 125 μL of water. The mixture was incubated under stirring for 15 min at room temperature, and the nanoparticles were separated by centrifugation (16 000 g, 15 min). The concentration of unbound drug was determined spectrophotometrically using an Infinite M1000Pro microplate reader (Tecan, Switzerland).

Animal experiments were approved by the Institutional Animal Care and Use Committee of the Shemyakin-Ovchinnikov Institute of Bioorganic Chemistry, Russian Academy of Sciences (protocol 367/2022 from 15.12.2022). Female BALB/c mice of 18–22 g weight (10–12 weeks old) were obtained from the Pushchino Animal Facility (Pushchino, Russia) and maintained under conventional conditions in an animal housing facility at the Institute of Bioorganic Chemistry, Russian Academy of Sciences, with a controlled light/dark cycle (12 h/12 h), temperature (22°C), humidity (50%), and unlimited access to dry food and water. Mice were divided into groups of 3–4 mice for testing coated and uncoated particles. Nanoparticles were injected into mice at a dose of 200 μg (10 mg/kg) into the retro-orbital sinus. At various time points, 20 μL of blood was collected from the opposite retro-orbital sinus and mixed with 1 μL of heparin. One hour after administration, the mice were sacrificed, and their major organs (liver, spleen, lungs, heart, kidneys, and femur bones) were isolated and weighed. Tissue and blood samples were digested in 3-fold volume of concentrated nitric acid at 60°C for 1 h, then diluted 7-fold with water and centrifuged at 20 000 g for 10 min. The Zr concentration in the samples was quantified by inductively coupled plasma mass spectrometry (ICP-MS) using a NexION 2000 mass spectrometer (PerkinElmer, USA). Biodistribution values were normalized to the total Zr content detected in all analyzed organs.

Statistical comparisons were performed using Welch’s *t*-test. *—p < 0.05. Data are presented as mean ± standard deviation. Hydrodynamic sizes and ζ-potentials of nanoparticles are reported as mode ± half-width at half-maximum.

## RESULTS AND DISCUSSION

**Fig. 1.**
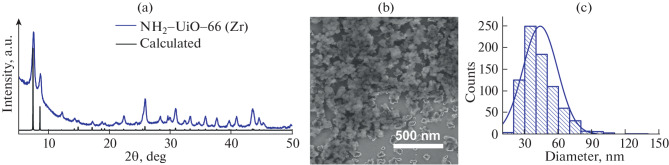
Characterization of NH_2_-UiO-66 (Zr) nanoparticles. (a) X-ray diffraction pattern of the synthesized MOFs compared to the calculated pattern of the UiO-66 crystal. (b) Scanning electron micrograph of the nanoparticles. Scale bar—500 nm. (c) Histogram of distribution of nanoparticles by size.

Nanosized metal-organic frameworks (MOFs) of the NH_2_-UiO-66 (Zr) crystal type were synthesized by a hydrothermal method in a microwave reactor. To obtain nanosized MOFs, acetic acid was added as a synthesis modulator to the reaction mixture containing the Zr (IV) precursor and 2-aminoterephthalic acid as the organic linker. Monocarboxylic acids can compete with the organic linker for coordination with the metal cluster, thereby slowing down the crystal growth. The crystalline structure of the nanoparticles was confirmed by X-ray diffraction (Fig. 1a). The powder diffraction pattern of the nanoparticles exhibited all the characteristic peaks of the UiO-66 framework, with a minor amorphous background [13], confirming the formation of a highly crystalline material. Based on the peak positions at 7.5° for the (111) plane, 8.5° for the (002) plane, 25.8° for the (006) plane, and 30.8° for the (117) plane, the synthesized crystals were determined to have a face-centered cubic topology with the Fm3m space group. Scanning electron microscopy revealed that the particles have irregular morphology (Fig. 1b). Size analysis based on measurements of more than 500 individual nanoparticles (Fig. 1c) showed that NH_2_‑UiO-66 (Zr) had a size of (44 ± 15) nm. The small particle size is consistent with rapid nucleation under microwave conditions with the growth moderated by the presence of acetic acid.

NH_2_-UiO-66 (Zr) nanoparticles lost their colloidal stability under physiological conditions simulated by 0.1 M phosphate-buffered saline (PBS, pH 7.4). In distilled water, the hydrodynamic diameter of NH_2_-UiO-66 (Zr) particles was (149 ± 41) nm, but it increased to (312 ± 82) nm after 5 min of incubation in PBS. To prevent particle aggregation, the nanoparticles were coated with silane-polyethylene glycol polymer using the Stöber reaction in aqueous conditions. After the coating, the ζ-potential of the nanoparticles decreased from (+35 ± 6) mV to (+12 ± 4) mV, consistent with surface modification by a neutral polymer. In addition, polymer coating led to a slight increase in hydrodynamic size of nanoparticles in water to (161 ± 52) nm. Surface modification of the MOFs with polymer drastically improved their colloidal stability, with the hydrodynamic diameter of NH_2_-UiO-66 (Zr)@PEG particles of (174 ± 52) nm after incubation in PBS.

The degradation and aggregation of NH_2_-UiO-66 (Zr) in PBS can be explained by the strong affinity of Zr (IV) clusters to phosphate groups and by the neutralization of the nanoparticle surface potential [14]. The presence of long polyethylene glycol chains (5 kDa) on the particle surface likely not only reduces the interaction of phosphates with the surface but also slow down their permeation into the porous structure. For example, previous studies showed that attachment of a shorter PEG chains (2 kDa) to UiO-66 (Zr) nanoparticles did not prevent aggregation under physiological conditions, although it still slowed down material degradation [15].

A coated NH_2_-UiO-66 (Zr)@PEG nanoparticles demonstrated the ability to be loaded with doxorubicin upon incubation in a drug solution. The loading efficiency was (1.6 ± 0.9)%, calculated as the drug mass normalized to the nanoparticle mass. The relatively low adsorption capacity is attributed to the large size of doxorubicin molecule (linear dimension ~15 Å), which enables interaction primarily with surface and structural defects of the nanoparticles. More efficient loading of chemotherapeutic agents into the particle volume [6, 7] is possible in the case of using small molecules such as 5-fluorouracil (∼5 Å [16]) and cisplatin (∼6 Å [17]), which linear dimensions are smaller than the dimensions of regular pores in the UiO-66 (Zr) structure and correspond to the dimensions of triangular windows (5−7 Å) in the pores [18]. However, this loading level is consistent with previous reports for similar metal-organic frameworks and is sufficient for effective therapy of cancer cells [8].

**Fig. 2.**
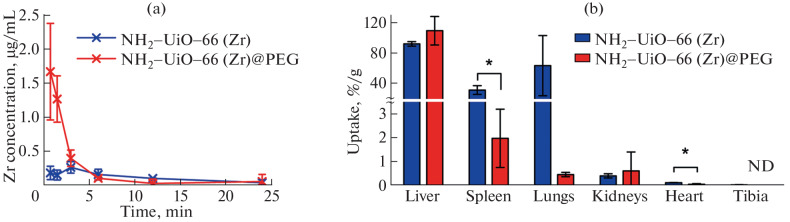
Blood circulation kinetics and biodistribution of uncoated NH_2_-UiO-66 (Zr) nanoparticles and polymer-coated NH_2_-UiO-66 (Zr)@PEG nanoparticles. * *p* < 0.05, Welch’s t-test. ND—not detected.

Then we investigated the effect of polyethylene glycol coating on the pharmacokinetics of nanoparticles after intravenous administration in healthy BALB/c mice. Uncoated NH_2_-UiO-66 (Zr) particles were almost completely cleared within the first minute post-injection (Fig. 2a), with Zr levels in the blood detected below 0.25 μg/mL level. In contrast, coated NH_2_-UiO-66 (Zr)@PEG nanoparticles exhibited typical exponential clearance kinetics, with a peak concentration of ~1.7 μg/mL. The total circulation time of  NH_2_-UiO-66 (Zr)@PEG particles was 12 min (Fig. 2a).

Surface PEGylation significantly altered the biodistribution of the nanoparticles (Fig. 2b). PEG coating increased hepatic uptake from 92 ± 3%/g to 110 ± 20%/g, while splenic accumulation decreased from 31 ± 6%/g to 2 ± 1%/g, and pulmonary accumulation decreased from 60 ± 40%/g to 0.4 ± 0.1%/g. The high accumulation of uncoated NH_2_-UiO-66 (Zr) nanoparticles in the lungs may be attributed to their aggregation and trapping in small pulmonary capillaries [10, 19]. After PEGylation of nanoparticles, accumulation in the following organs did not change significantly: kidneys change from 0.4 ± 0.1%/g to 0.6 ± 0.8%/g; heart—from 0.11 ± 0.01%/g to 0.04 ± 0.03%/g; and bone tissue—from 0.017 ± 0.004%/g to undetectable levels. Therefore, surface modification with PEG substantially improved the pharmacokinetics of NH_2_-UiO-66 (Zr) MOFs, preventing their aggregation, prolonging circulation time, and reducing nonspecific accumulation in lung capillaries and spleen.

## CONCLUSIONS

In summary, here we synthesized nanosized metal-organic frameworks NH_2_‑UiO‑66 (Zr) with polyethylene glycol coating. The obtained MOFs demonstrated colloidal stability in phosphate-buffered saline and the ability for doxorubicin loading. This enables to perform drug delivery after systemic intravenous administration. Preliminary pharmacokinetic studies of NH_2_‑UiO‑66 (Zr)@PEG nanoparticles in healthy mice showed that PEGylation of surface markedly reduced nonspecific accumulation of nanoparticles in highly vascularized organs, such as the lungs and spleen.
